# Inactivation and Elimination of Centrioles During Development in the Genus *Drosophila*: Current Insights and Open Questions

**DOI:** 10.3390/cells14120865

**Published:** 2025-06-08

**Authors:** Denise Bonente, Giuliano Callaini, Maria Giovanna Riparbelli

**Affiliations:** Department of Life Sciences, University of Siena, Via A. Moro 2, 53100 Siena, Italy; denise.bonente2@unisi.it (D.B.); riparbelli@unisi.it (M.G.R.)

**Keywords:** centriole, centrosome, inactivation, elimination, *Drosophila melanogaster*

## Abstract

Centrioles are remarkably stable organelles that play a main role in the assembly of centrosomes and ciliary structures. However, there are several differentiated tissues that must eliminate their centrioles to avoid centrosome formation and improper cell proliferation. Therefore, centriole elimination represents an important process in many organisms to ensure successful cell differentiation and maintenance of tissue homeostasis. In this review, we analyzed centriole inactivation and elimination in various *Drosophila* cell types in relation to the dynamics of the pericentriolar material.

## 1. Introduction

Centrioles are widely conserved barrel-shaped organelles composed of triplet, doublet, or single microtubules, usually arranged in a beautiful ninefold symmetry [1,2,3,4,5]. Centrioles are essential for proper cell function: they are found at the focus of the centrosome, the nucleating center for the microtubule network in interphase and cell division, and represent the basis of the ciliary structures involved in cell movement and cell signaling [6,7,8,9]. Therefore, centriole dynamics are crucial for cell homeostasis [10]. Since centrioles represent the core components of centrosomal proteins such as centrosomin (Cnn), spindle defective 2 (Spd2), and γ-tubulin, which are directly involved in the nucleation of the microtubule network, their duplication must be strictly regulated during the cell cycle to avoid too many centrosomes [11,12,13]. Centriole assembly and replication in cycling cells occur during interphase and rely on a set of core proteins forming the so-called Plk4-Stil-Sas6 module [14,15,16,17,18].

Despite the need for centrioles to organize a functional centrosome able to nucleate the network of cytoplasmic microtubules in interphase and during mitosis [19], there are several examples of differentiating cells that eliminate or inactivate their centrioles [20]. The most remarkable example is the *Drosophila Spindle assembly abnormal 4 (Sas4)* mutant in which adults eclose in the absence of centrioles [21], suggesting that centrioles can be dispensable in some cases for development. However, the centrioles are needed in *Sas4* embryos to sustain early development until the beginning of gastrulation when the maternal supplied factors rapidly decrease [22].

Centriole elimination is important to warrant the correct homeostasis of some differentiated tissues. There are several instances in which the loss of centriole integrity or defective centriole/centrosome biogenesis critically contributes to specific diseases, including cancer, ciliopathies, and developmental disorders [23,24,25,26,27,28]. Furthermore, the failure to eliminate centrioles does not prevent the generation and does not alter the fate of supernumerary centrioles in dividing cells [29]. Aneuploidy, which is a hallmark of cancer cells, correlates with the presence of extra centrosomes, which originate from centriole overduplication [13,30,31].

The elimination of the centrioles is a remarkable phenomenon, considering the high stability of this organelle and its persistence within the conditions that lead to microtubule depolymerization. However, despite extensive knowledge of the molecular players involved in centriole assembly, it is still unclear how centriole stability is maintained, and the mechanisms leading to centriole elimination remain an important challenge.

Various *Drosophila* cell types lack functional centrosomes and organize specialized microtubule networks by non-centrosomal microtubule organizing centers (ncMTOCs) [32]. Non-centrosomal microtubule arrays have been described in *Drosophila* during oocyte development [33], in muscle cells [34], within dendrite branches [35], along the mitochondrial derivatives during spermatid elongation [36,37], and around the nuclear envelope of larval fat body cells [38]. The lack of functional centrosomes is typical of postmitotic polyploid cells such as salivary glands, midgut, and Malpighian tubule cells [39], although it is still unclear the relationship between the multiple rounds of chromosome replication and the absence of true centrosomes.

Replacement of functional centrosomes with ncMTOCs in *Drosophila* occurs through an unusual centriole dynamic. Early differentiating ovarian follicle cells [40], salivary gland cells [41], tracheal cells [42], wing epithelial cells [43,44], and ommatidial cells [45,46,47] first assemble a microtubule network by centrosome-dependent mechanisms. Later, however, the centrosome is inactivated, and its core centrioles no longer localize near the new microtubule nucleating foci. Centriole reduction or defects in their structure have been reported in *Drosophila* after mutations in genes encoding proteins that regulate or build the centriole architecture [48]. These findings suggest that *Drosophila* could represent a powerful system in which to examine the reduction in centrioles during development and differentiation, and it may be a helpful model to uncover the structural changes leading to centrioles elimination.

**Figure 1:**
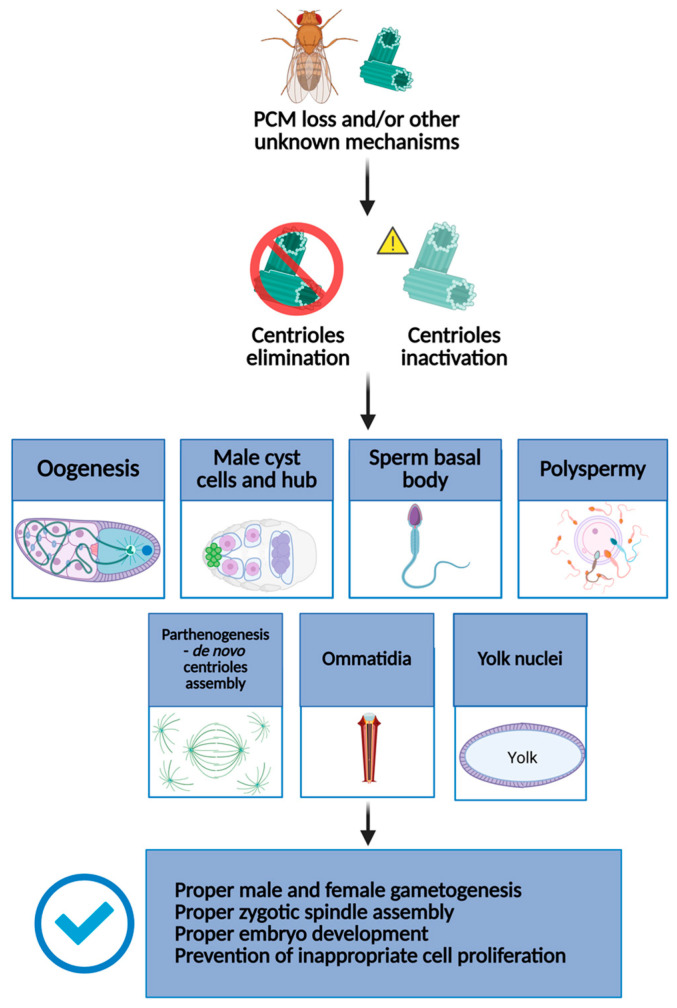
Schematic representation of how, in *Drosophila*, centrioles are eliminated or inactivated at key stages of development and cellular differentiation. In particular, centrioles are eliminated or inactivated during oogenesis, spermiogenesis, and parthenogenesis during the early stages of embryo development. Centrioles are also inactivated in differentiated cells, such as ommatidia. These processes are essential to prevent aberrant cell divisions, ensure proper gametogenesis, and support normal embryonic development. Created in BioRender, https://BioRender.com/37eu8sg (accessed on 21 May 2025).

The individual examples described below illustrate that there is a subtle diversity in how *Drosophila* cells ensure centriole elimination or inactivation, suggesting that the mechanisms driving these processes could be slightly different through various tissues (Figure 1).

## 2. The Stem Cell Niche of the Drosophila Testis: Somatic and Germline Centrioles with Different Fates

The apical region of the *Drosophila* testis contains a niche with a cluster of small somatic cells, the hub cells, surrounded by 8–10 germline stem cells (GSCs) that will be the progenitors of the male gametes. During spermatogenesis and spermiogenesis, each GSC is surrounded by a pair of somatic cyst stem cells (CySCs) that are progenitors of the thin cyst cells [49]. The asymmetric division of each GSC leads to a new stem cell and a gonioblast daughter, which progress through spermatogenesis to form the haploid spermatids that will differentiate in mature sperms. The CySCs also undergo asymmetric divisions to generate new CySCs and cyst cell daughters that withdraw from the cell cycle and will modify their size and shape, becoming very flat and large.

Centrioles in germ cells and cyst cells differ in their functional abilities and timing of activity in accordance with the behavior of their respective cell types. Centrioles of germ cells recruit pericentriolar material (PCM) to assemble functional centrosomes needed to warrant spermatogonial mitosis and spermatocyte meiosis. In addition, the centrioles act as basal bodies to nucleate the axoneme of the cilium-like regions (CLRs) of the spermatocytes and the flagellum of the differentiating sperm cells. Conversely, the cyst cells become quiescent, and their centrioles gradually lose the ability to recruit PCM, including γ-tubulin, but keep the cartwheel component Sas6. This suggests that centrioles are present but inactivated. Indeed, the cyst cells have centriole pairs, at least during the first spermatogonial mitosis, but the daughter centriole is very short and does not elongate further [50], pointing to a correlation between centriole growth and availability of centrosomal material. Similarly, the daughter centriole that remains in the ooplasm at the end of meiosis in the starfish *Patiria miniata* sheds the surrounding PCM and then disappears [51]. Moreover, centriole duplication does not occur in cyst cells, possibly due to the downregulation of Sas6, a main regulator of centriole architecture [50]. The failure of centriole duplication represents an important limitation since cyst cell division could compromise the integrity of the entire germ cell, impairing the correct progression of gametogenesis.

The other class of somatic cells building the stem cell niche are the small post-mitotic cells of the hub. Centrioles are occasionally found in the hub cells, but they are single and lack procentrioles, suggesting failure to duplicate. Moreover, γ-tubulin, pericentrin-like protein (Plp), and spindle defective 2 (Spd2), usually observed in GSCs and CySCs, are barely detectable or absent in the hub region [52,53]. The failure to recruit these centrosomal proteins confirms that the rare hub centrioles are not functional. The inactivation of the centriole function is an intrinsic property of the cyst cells, and it is unrelated to the germ cells they surround. Indeed, CySCs of the *Drosophila* mutant *nup154* [54] undergo asymmetric division even in the absence of GSCs, and the daughter cyst cells are unable to experience mitosis, and their centrioles do not organize functional centrosomes. However, although hub cells are generally considered terminally differentiated and quiescent, some observations suggest that they can exit quiescence to generate new CySCs [55]. This raises the intriguing question of how these cells regain centrosome function to re-enter the cell cycle and proliferate.

The centrioles of the hub cells have a remarkable architecture and consist of mixed doublet and triplet microtubules. This condition questions the general opinion that *Drosophila* flies would display two distinct and separate centriole models: centrioles with nine microtubule doublets, only found in somatic cells, and centrioles with nine microtubule triplets typical of male and female germline cells (see [56,57]). The mixed doublet and triplet microtubules of the hub cell centrioles suggest that the acquisition of the C-tubule could sporadically occur in the stem cell niche.

The pole cells, the precursors of the germ cell line, have centrioles with doublet microtubules, like somatic cells, and the GSCs acquire the C-tubule before their asymmetric division [52]. But if it is so, many questions remain open: what kind of information is required to acquire the C-tubule? Why do the hub cells not complete the triplet pattern? Why do the CySCs, located in the stem cell niche, have centrioles with only doublet microtubules?

In addition, the centrioles of the hub cells lack a distinct cartwheel, and the centrioles of the cyst cells show slight signs of degeneration. Remarkably, centriole elimination during *C. elegans* oogenesis begins with the loss of the central tube, followed by the disassembly of microtubules. [58]. However, the cartwheel, which appears to be a constant structure of *Drosophila* somatic and germ cell centrioles, lacks both the tandemly aligned centrioles that are at the basis of the olfactory neurons. The distal centriole that nucleates the ciliary axoneme of the olfactory neurons is made up of nine doublets of microtubules, whereas the wall of the proximal centriole consists of both doublet and single microtubules. The single microtubules often display a lateral hook, suggesting that the proximal centrioles grow incompletely or, alternatively, that they are at the beginning of their degradation.

Moreover, the proximal centrioles, despite their incomplete architecture, recruit some typical centrosomal components such as Plp, Anastral spindle 1 (Ana1), Spd2, and γ-tubulin [59] and are exceptionally stable throughout the life of the fly. This suggests that the incomplete wall of the proximal centriole in the olfactory ciliated neurons does not necessarily correlate with decreased function or stability.

A noteworthy structural asymmetry between the centriole pair has been reported in the mature mammalian sperm. However, contrary to the centrioles of the *Drosophila* olfactory neurons, the distal centriole that templates the flagellar axoneme degenerates during human spermiogenesis, whereas the proximal inactive centriole is structurally unchanged [60,61].

## 3. Oogenesis: The Main Example of Centriole Elimination

The formation of the zygotic spindle in most organisms usually requires the contribution of the male gamete that provides its basal body at fertilization. Indeed, the basal body functions as a true centriole, recruiting all the components from the oocyte cytoplasm required to organize a functional centrosome capable of nucleating the microtubules that support pronuclear migration. After the pronuclei come into contact, the centrosome duplicates to assemble the bipolar zygotic spindle. Thus, the assembly of the zygotic spindle requires the presence of only a single centrosome at fertilization since multiple centrosomes can nucleate supernumerary astral microtubules that can have deleterious effects on embryonic development. Therefore, the egg must be centriole-free to avoid too many centrosomes at fertilization and ensure that only the sperm basal body can act as the master for the zygotic spindle assembly. The oocyte coming from transit-amplifying oogonial mitotic divisions must eliminate or inactivate any inherited centrioles/centrosomes upon entry into meiosis. Thus, centriole elimination during oogenesis is a widespread phenomenon that occurs throughout metazoan organisms. There are two striking examples of centriole elimination during female meiotic progression: the case of echinoderms and the case of insects and mammals [62]. Echinoderms maintain centrioles, and the meiotic spindles are organized by true centrosomes that progressively reduce their centriole content. Thus, the spindle poles of the first meiotic spindle consist of a centriole pair, whereas during the second meiosis, only one centriole is found at each pole. The extrusion of the last polar body results in the inheritance of the daughter centriole in the egg cytoplasm. However, the daughter centriole is unable to sustain microtubule nucleation and cannot interfere with embryonic development [51]. Conversely, mammalian and insect oocytes progress through meiosis without centrioles, and their spindles are anastral. The fate of centrioles in the *Drosophila* oocyte is particularly noteworthy [63]. *Drosophila* oogenesis begins in the anterior region of the ovariole, where two to three GSCs divide asymmetrically to self-renew and give origin to daughter cystoblasts that undergo four incomplete mitotic divisions to form cysts of 16 germ cells interconnected by cytoplasmic bridges, or ring canals [64]. Each of the 16 germ cells inherits a pair of centrioles consisting of nine triplet microtubules. Early in oogenesis, when the oocyte starts to differentiate, the centrioles travel through the ring canals of the sister cells and cluster in the posterior region of the oocyte cytoplasm [65]. The mechanisms driving the movement of each centriole pair from the nurse cells toward the oocyte cytoplasm are still poorly understood. The centrioles are inactive and do not organize functional centrosomes during migration, but a large MTOC appears when the centrioles cluster at the posterior region of the oocyte [66]. Centrioles have been observed at the ultrastructural level in the oocyte cytoplasm during mid-oogenesis [33], and typical centriole markers have also been observed during later stages of oogenesis [67]. Immunofluorescence analysis of the centriolar components Ana1 and Sas6 support the gradual diminution through oogenesis of the centriole number and their loss just before meiotic spindle assembly [67]. The problem here is how the centrioles are eliminated to ensure the correct centriole number upon fertilization. It has been suggested that centriole elimination could be a consequence of the loss of the PCM and that Polo loss could be a critical event in triggering the loss of the PCM, followed by centriole elimination. Indeed, downregulating Polo expression accelerates centriole loss, whereas ectopic tethering of Polo to centrioles prevents the loss of PCM, inhibiting the elimination of the oocyte centrioles [67]. The Polo-induced stability to the oocyte centrioles leads to abnormal meiosis and aborted embryonic development.

Ultrastructural observations of the centriole cluster at the posterior region of the oocyte cytoplasm revealed centrioles with different lengths and centrioles with slight signs of fragmentation.

## 4. The Sperm Centriole: An Immortal Contribution?

Successful embryonic development in most organisms requires the contribution of the sperm cell that supplies its nucleus and the basal body, with the latter being involved, during spermiogenesis, in the assembly of the axoneme, which gives motility to the male germ cell. The axoneme and the basal body usually separate in the ooplasm of many animals [68,69], presumably to facilitate the role of the inherited centriole as organizer of the functional zygotic centrosome. The elimination of the sperm tail is, indeed, vital to early development in mammals [70], and the persistence of the tail near the zygotic spindle results in developmental failures of the human embryo [71].

The whole sperm cell enters the *Drosophila* oocyte at fertilization [72,73,74], and the sperm tail, about 2 mm in length, was seen within the yolk region during the blastoderm stage [75] and persisted into the larval midgut after hatching [76]. Remarkably, the sperm tail ends at one mitotic spindle during the nuclear divisions of the early syncytial *Drosophila melanogaster* [77] and *Drosophila simulans* [78] embryos. This suggests that the sperm basal body is not excised, but both the structures are intact and remain associated through early development. Indeed, a giant centriole was seen at the apical end of the sperm axoneme [79], confirming a continuity between the sperm axoneme, the centriole, and the mitotic spindle poles. The sperm centriole recruits PCM, and it is found at the core of the true centrosome that organizes the microtubule aster, needed for the apposition of the parental pronuclei, and then it associates with one pole of the zygotic spindle. This process is in apparent contrast with the finding in vertebrate cells that the mother centriole, involved in primary cilium assembly, must shed axonemal microtubules to leave the cell surface and move inward to organize a functional centrosome. However, during insect spermatogenesis, both parental centrioles form microtubule-based CLRs that persist during the assembly of the meiotic spindles [80]. The sperm centriole, despite its link to the axoneme, can duplicate but does not form a mirror image. The daughter centriole is, indeed, smaller than the mother and consists of nine doublets of microtubules, as the ensuing centrioles of somatic cells (and as well all the somatic centrioles that derive from it), while the sperm centriole maintains nine triplets of microtubules.

The two main open questions here are the following: how long is the life of the sperm centriole during development, and how long is its ability to assemble functional centrosomes? The sperm centriole and its tail dissociate from the surface nuclei before cellularization and seek inward. This process is apparently correlated with the reduction in the ability of the sperm centriole to recruit PCM. The amounts of centrosomin (Cnn) and Spd2 slightly reduce when the sperm centriole is at the embryo surface and continues to decrease during early development. A feeble Spd2 labeling is found at the beginning of gastrulation when the sperm centriole is within the yolk region, whereas no more Cnn signal is seen at this stage [79].

Therefore, the first sign of basal body inactivation is the gradual loss of centrosomal material. Presumably, the loss of pericentriolar components could ultimately lead to centriole degradation. This is consistent with the idea that the PCM is needed to warrant centriole duplication and maintenance (integrity, stabilization) in *Drosophila melanogaster* [67,81,82,83].

## 5. How Many Centrioles at Fertilization? The PCL Hypothesis

Centriole duplication is accurately monitored during cell division to avoid too many active centrosomes that may have detrimental effects. This process in somatic cells is tightly coupled to DNA replication [84]. However, during male gametogenesis in the majority of animals, mammals included, centriole duplication also occurs during the second meiotic division in the absence of DNA replication. Thus, the haploid spermatids inherit a pair of centrioles that are transferred to the oocyte at fertilization. This is not the rule during insect spermatogenesis in which the centrioles duplicate in primary spermatocytes, according to DNA replication, but fail to duplicate during the second meiosis [56]. Although it appears trivial that the mature sperm carries only its basal body at fertilization, it is still unclear if the sperm cell can also contribute an atypical unstructured procentriole. This conundrum arises from the observation that typical centriolar proteins, namely Ana1, Bld10, Sas6, and Asterless (Asl), are detected as a small dot-like signal in the so-called proximal centriole-like (PCL) structure close to the basal body of early elongating *Drosophila* spermatids [85,86,87]. These findings point to the presence of a PCL organelle [85]. Ultrastructural observations of early elongating *Drosophila* spermatids have, indeed, revealed the presence of an unusual procentriole consisting of a distinct cartwheel and an incomplete set of single peripheral tubules, presumably the A-tubules [52]. Remarkably, co-overexpression of the centriolar proteins Sas6 and Poc1B, which are enriched in the PCL [88], induces the formation of ectopic aggregates containing PCL-like structures [89].

The dot-like signals of the centriolar proteins disappear during spermiogenesis [90], and a structured PCL is no longer found in mature sperm [52]. This opens questions on the transient nature and function of the PCL. It has been proposed that the PCL may contribute to the correct anchoring of the basal body to the nuclear envelope during sperm elongation [91] and may represent a focus for the assembly of the second centriole during the formation of the first zygotic spindle [90]. However, it is unclear why the procentriole appears at the onset of spermiogenesis when centriole duplication represents an unexpected and unusual feature for centriole biogenesis.

Distinct procentrioles have also been observed close to the basal body of young spermatids in the coleopteran *Adalia* [92] and *Tribolium* [93]. Interestingly, the procentriole of *Adalia* looks like the one of *Drosophila* and consists of a cartwheel and peripheral single microtubules, whereas the procentriole of *Tribolium* lacks microtubules but only displays a dense peripheral ring.

Similar intermediate procentriole-like structures, consisting of a distinct cartwheel and peripheral single microtubules, have also been observed in the lepidopterans *Bombix* [94], *Ephestia* [95], and *Pieris* [96]. These structures, in clusters of three to four, appear before spermiogenesis and are observed in mature primary spermatocytes close to the still-engaged parental centrioles. However, young *Pieris* spermatids maintain only one procentriole that is no longer detected in mature sperm.

Thus, the male insect gamete could provide at fertilization one true centriole and an unstructured, undisguisable precursor that could act as a scaffold for the rapid assembly of a second zygotic centriole [97]. The problem here is why and how the structured procentriole is eliminated during sperm maturation and what residue, if any, the sperm carries into the egg at fertilization. It is also still unclear how the unstructured precursor can be converted into a functional centriole at fertilization.

## 6. Polyspermy: Elimination of Redundant Sperm Centrioles

The apposition of male and female pronuclei at fertilization requires the microtubule aster nucleated by the centrosome that is reconstituted around the inherited sperm centriole. Therefore, only one sperm is needed to warrant the successful fusion of the parental pronuclei. Multiple sperm can provide extra centrioles, leading to the formation of supernumerary asters with ensuing developmental defects. Most dioecious animals have developed various strategies to prevent excess sperm from entering the egg and avoid abortion of the first embryonic divisions. However, animals that lay large eggs allow the entry of numerous sperm without detrimental effects on the development of the embryo [98,99]. This process, called physiological polyspermy, which increases the chance that a sperm will meet its female counterpart in a very large cytoplasm, raises intriguing questions about how a single sperm is selected while the others are discarded. It is also unclear how only one centriole organizes the zygotic centrosome, whereas the other will be eliminated.

The entrance of more than one sperm into the egg is a rare event during insect fertilization [100]. Only in a few cases have multiple sperm been observed in the egg cytoplasm of *Drosophila melanogaster* [63], and supernumerary paternal nuclei have only occasionally been found [101]. Remarkably, *Drosophila obscura* embryos complete gastrulation with multiple sperm [75]. Although monospermy is the rule in *Drosophila melanogaster* [102], the few cases in which more than one sperm is present at fertilization raise the question of how supernumerary sperm do not affect embryo development.

Some models have been proposed during urodele amphibian fertilization to explain the selection of one sperm nucleus and the elimination of the supernumerary sperm [99]. Among these models, the failure to enter M-phase could explain the degeneration of the accessory sperm nuclei [99]. Although appealing, this hypothesis does not seem to be applicable in the few cases of *Drosophila* polyspermy, in which the sperm nuclei are arrested in an anaphase-like configuration [101]. Here, the haploid chromosomes are congressed at the metaphase plate, and the sister chromatids separate at the onset of anaphase but do not migrate to the opposite poles, pointing to defects in the mitotic spindle architecture and dynamics. Each pole of the anaphase-like spindles displays a pair of centrosomes nucleating short astral microtubules, whereas the anaphase zygotic spindles that hold both male and female complements had only one centrosome for each pole. It is unclear whether each centrosome of the pair contains a typical pair of centrioles. If this was the case, we can hypothesize that centriole duplication occurs despite the fact that the chromosomes do not duplicate. Presumably, the centrosomes detach from the abnormal spindles and subsequently degenerate.

## 7. Parthenogenesis: Too Many Centrioles Do Not Affect the Embryonic Development

Although the centrioles appear to be useless organelles during oogenesis, they must be eliminated because their retention is deleterious for embryonic development. Centriole elimination during oogenesis is needed to ensure the correct centriole number upon fertilization; thus, the mature oocyte usually lacks centrosomes. Some parthenogenetic insects that develop without male contribution, such as the collembolan *Folsomia candida* [103], the viviparous pea aphid *Acyrthosiphon pisum* [104], the parasitic hymenopterans *Nasonia vitripennis* [105] and *Muscidifurax uniraptor* [106], and the dipterans *Drosophila mercatorum* [107,108] and *Drosophila ananassae* [109] also eliminate the maternal centrioles during oogenesis, and the meiotic spindles are anastral. However, a variable number of microtubule asters appeared in the oocyte cytoplasm after anaphase of the first meiosis. Ultrastructural observations revealed that the focus of the cytoplasmic asters contains distinct centrioles [110], supporting the de novo assembly of these organelles despite the absence of pre-existing templates. The presence of cytoplasmic asters appeared as a conserved feature shared among all the insect species until now examined and represents the basis for the assembly of the first zygotic spindle.

Although it is still unclear how the haploid female pronucleus resumes diploidy during parthenogenetic development, we noticed that all the collembolan, aphid, and hymenopteran parthenogenetic eggs develop properly, whereas only 8–10% of the eggs laid by the *Drosophila* parthenogenetic species eclose, pointing to a stochastic phenomenon rather than to the presence of operative mechanisms. Therefore, the parthenogenetic development of *Drosophila mercatorum* and *Drosophila ananassae* appears as a recently acquired process.

A common feature of all the parthenogenetic eggs is the persistence of several astral microtubules during embryonic development, suggesting that the supernumerary centrioles can successfully recruit centrosomal proteins. As discussed above, one main problem at fertilization is the control of the centriole number since the zygotic spindle only needs two pairs of centrioles to be assembled. Thus, the simultaneous presence of several asters within the egg cytoplasm raises the question of how the egg can properly organize the first bipolar spindle and undergo further mitotic divisions without the negative interference of the supernumerary centrioles. Consequently, most animal eggs develop different strategies to avoid supernumerary centrioles at the beginning of embryo development. Such controls prevent the assembly of multipolar spindles and the ensuing developmental defects that are seen during polyspermic fertilization in which multiple sperm asters result in aberrant spindles and developmental failure [68,100]. However, parthenogenesis occurs in a condition resembling polyspermy, but the supernumerary asters do not have a deleterious effect on development and are prevented from interacting with the zygotic spindle.

Indeed, in Aphids and Hymenopterans, only one or two asters contact the female pronucleus, whereas, in the *Drosophila* species with occasional parthenogenesis, multiple asters may interact with the chromatin, leading to abnormal spindles and developmental failures [107,108,109]. This indicates that a mechanism limiting aster interaction with the female pronucleus does not work in *Drosophila*. Moreover, the few *Drosophila* embryos that develop parthenogenetically have many free cytoplasmic asters, mainly localized in the anterior region of the embryo.

Remarkably, the multiple centrosomes in the *Drosophila* parthenogenetic eggs seem to duplicate in a cell cycle-dependent manner: single asters have been observed during interphase, whereas distinct pairs are found in prometaphase. Close aster pairs are also visible during late anaphase/telophase, the time the centrioles disengage at the spindle poles. These observations suggest that centriole duplication is unrelated to DNA replication and involves an unknown cytoplasmic clock. Interestingly, it has been demonstrated in *Drosophila* eggs that centrioles form de novo and duplicate at an elevated Polo-like kinase 4 (Plk4) concentration [111]. This suggests that Plk4 concentration determines the temporal onset of centriole assembly.

Unfortunately, the molecular mechanism triggering centriole de novo assembly at anaphase of the first meiosis remains largely unknown. It is known that overexpression of typical centriolar proteins, such as Plk4, Sas4, Sas6, and Asl, leads to de novo assembly of centrioles in *Drosophila* unfertilized eggs [112,113,114]. However, a correlation between the overexpression of these proteins and parthenogenetic development needs to be demonstrated.

The asters disappear at different times in different parthenogenetic species. *Muscidifurax uniraptor* embryos lose their asters during the third mitosis. By contrast, *Drosophila mercatorum*, *Drosophila ananassae*, and *Acyrthosiphon pisum* embryos retain some asters until later cleavage divisions. This suggests that different insect species exploit different mechanisms to inactivate and eliminate supernumerary centrosomes from the cytoplasm of the developing embryo. The mechanisms underlying centriole elimination at different times are still unclear and need further investigation and careful ultrastructural analysis.

## 8. Yolk Centrosomes: Different Fates in the Same Cytoplasm

The development of the early *Drosophila* embryo consists of 13 nuclear divisions occurring in a common syncytium that later cellularizes to form a surface blastoderm of about 6000 cells. The first nuclear divisions occur in the central region of the egg, but during telophase of the eighth and ninth mitoses, the majority of the nuclei migrate towards the cortical region of the embryo, whereas other nuclei remain stationary in the inner cytoplasm.

Thus, two distinct nuclear lineages with different fates segregate at the end of the ninth mitotic nuclear division: the peripheral blastodermic nuclei that will be the precursors of the somatic tissues and the inner nuclei that are involved in yolk digestion [115] and midgut development [116]. An unusual and remarkable feature is that each of the nuclear lineages has its own division program despite all residing in a common cytoplasm. The peripheral nuclei undergo four additional mitotic divisions to give rise to a syncytial blastoderm that later cellularizes [117]. By contrast, the yolk nuclei undergo two abnormal divisions followed by two rounds of DNA replication without karyokinesis [77].

The centrosomes associated with the peripheral nuclei organize functional mitotic spindles that support chromosome congression at the metaphase plate and successful chromatid migration to opposite spindle poles. By contrast, the centrosomes in the inner cytoplasm, hereafter called yolk centrosomes, display unusual dynamics. The yolk centrosomes organize normal bipolar spindles during the nuclear cycle 10, but starting from nuclear cycle 11, the majority of the yolk centrosomes duplicate but do not separate properly, and most of the spindles are monopolar. During nuclear cycles 12 and 13, most of the centrosomes detach from the spindle poles and are found free in the inner cytoplasm far from the abnormal chromatin clusters [118]. Remarkably, the free yolk centrosomes can duplicate further, and clusters of four or more centrosomes have been observed during the cellularization of the syncytial blastoderm and at the beginning of gastrulation. This suggests that the yolk centrosomes can duplicate regardless of their incomplete separation and in the absence of nuclear divisions, whereas the centrosome cycle in the cortical region of the embryo is strictly coupled to the nuclear divisions. It is, therefore, intriguing to speculate that centrosomes in the inner cytoplasm and in the cortical region respond to different requirements, although during the early nuclear division cycles, all centrosomes duplicate in synchrony.

The isolated yolk centrosomes recruit γ-tubulin after the nuclear cycle 12 but do not nucleate distinct microtubules, as they do during the previous nuclear cycles, although a small pool of free tubulin is associated with them. This implies a progressive inactivation of the yolk centrosomes that gradually lose their microtubule nucleating properties before disappearing during late gastrulation. However, the molecular mechanisms leading to the inactivation of the yolk centrosome and the elimination of the core centrioles are still unclear.

## 9. Ommatidial Differentiation: A Tale of Useless Centrioles

The *Drosophila* eye is formed by ∼750 ommatidial units derived from undifferentiated proliferating cells that form the eye-antennal disc [119,120,121]. The patterning of the ommatidia requires dramatic cell transformations that begin in early third-instar larvae when the eye disc is crossed by the morphogenetic furrow [122]. This structure, in the form of a deep indentation of the epithelium, represents the boundary between the anterior region of the disc where the undifferentiated cells proliferate and the posterior region in which the rhabdomeric cells differentiate to organize the ommatidial units [123,124,125].

The cells of the anterior region of the imaginal disc hold distinct centrosomes that assemble functional mitotic spindles. Such centrosomes, enriched in Cnn and Spd-2, actively recruit γ-tubulin [45,126]. Conversely, the cells of the posterior region of the imaginal disc do not proliferate, and their centrioles are unable to recruit the typical centrosomal proteins. Thus, in the absence of γ-tubulin, the centrioles of the post-mitotic differentiating rhabdomeric cells are not involved in the nucleation of the longitudinal bundles of microtubules that fill the whole cytoplasm [45,47]. Rather, γ-tubulin foci are found at the apical cell membranes, suggesting that they may represent non-conventional MTOC like those described in the cone cells of the *Drosophila* ommatidia [46]. In addition to the earlier failure of γ-tubulin recruitment, the centrioles of the rhabdomeric cells did not accumulate Cnn and Spd2 proteins during the late larval stages.

Remarkably, the centrioles of the pupal eyes gradually lost the scaffold protein Plp, whereas the core proteins Asl, Ana1, and Sas4 disappeared later, pointing to the progressive disappearance of the centrioles. Although the centrosomal proteins γ-tubulin, Cnn, Spd2, and Plp are lost during late larval and early pupal stages, the structure of the centrioles remains unchanged and displays the usual model of nine doublets of microtubules with a distinct cartwheel, suggesting that the depletion of the PMC does not affect the centriole architecture. However, during later pupal stages, the centriole wall displays doublet and single microtubules. Moreover, the A-tubule holds lateral short hooks, suggesting the progressive deletion of the B-tubule. A distinct cartwheel is no longer observed at this stage of development.

## 10. Conclusions

Centrioles are very stable organelles that maintain intact their structure during cell cycle progression and embryo development. However, some differentiating tissues eliminate or inactivate their centrioles to avoid the formation of unnecessary functional centrosomes and then prevent inappropriate cell proliferation. Therefore, an important aspect to clarify is to understand the mechanisms that enable centriole stability and the process leading to centriole disassembly. PCM removal plays a critical role in centriole stabilization in some *Drosophila* cells. Indeed, the continuous expression of cartwheel, centriole wall, and PCM components play a main role in ensuring centriole integrity. However, the relationship between the reduction in PCM components and centriole deconstruction is not a widespread phenomenon, as there are animal cells in which centriole stability is apparently unrelated to PCM persistence [127,128,129].

Although some data on the role of centriolar proteins and centrosomal components involved in managing centriole stability have led to suggestions of hypothetical mechanisms underlying stepwise centriole elimination [20], the structural changes leading to centriole disassembly are still poorly understood.

The few ultrastructural observations concerning the dynamics of centriole disassembly come from analysis of *C. elegans* oocytes [58] and embryos [20], *Naegleria gruberi* ameboid form [130], *Drosophila* mid-oogenesis, ommatidial differentiation and male stem cell niche [45]. These observations show that centriole elimination occurs according to different stereotypical manners in various model systems, suggesting different elimination programs.

In particular, the ultrastructural analysis in *Drosophila* points to a dismantlement of the centrioles by the gradual disappearance of the C- and B-tubules of the wall, depending on germ or somatic cells. The ultrastructural observations uncovering centrioles with signs of degeneration agree with the gradual reduction in the PCM associated with such centrioles. Remarkably, it has been demonstrated that the recruitment of Ana1 by RCD4 is sufficient to stabilize the microtubules triplets during male *Drosophila* gametogenesis [131], and the expression of Ana1 in the *Drosophila* oocyte is sufficient to prevent centriole disassembly during oogenesis [83]. Ana1, RCD4, Polo, and PCM components are indeed considered key factors contributing to centriole/centrosome maintenance [28].

Therefore, at least in some *Drosophila* cells, the gradual disappearance of the PCM components is associated with the reduced functionality of the centrosomes and then with the subsequent degeneration of the centrioles. However, there are *Drosophila* differentiated cells in which centrioles lost some of their PCM-associated components but persist longer. This suggests that the relationship between PCM reduction and centriole elimination is far from understood and requires further investigation.

## Abbreviations

The following abbreviations are used in this manuscript:

Ana1	Anastral spindle 1
Asl	Asterless
CLRs	Cilium-like regions
Cnn	Centrosomin
CySCs	Cyst stem cells
GSCs	Germline stem cells
ncMTOCs	Non-centrosomal microtubule organizing centers
PCM	Pericentriolar material
Plk4	Polo-like kinase 4
Plp	Pericentrin-like protein
Sas	Spindle assembly abnormal
Spd2	Spindle defective 2
